# Identification of Two Potential Gene Insertion Sites for Gene Editing on the Chicken Z/W Chromosomes

**DOI:** 10.3390/genes15070962

**Published:** 2024-07-22

**Authors:** Gaoyuan Wu, Youchen Liang, Chen Chen, Guohong Chen, Qisheng Zuo, Yingjie Niu, Jiuzhou Song, Wei Han, Kai Jin, Bichun Li

**Affiliations:** 1 Joint International Research Laboratory of Agriculture and Agri-Product Safety of Ministry of Education of China, Yangzhou University, Yangzhou 225009, China; mz120221506@stu.yzu.edu.cn (G.W.); adam3042705712@outlook.com (Y.L.); 13773520825@163.com (C.C.); ghchen0115@yzu.edu.cn (G.C.); 006664@yzu.edu.cn (Q.Z.); 007510@yzu.edu.cn (Y.N.); 2 Key Laboratory of Animal Breeding Reproduction and Molecular Design for Jiangsu Province, College of Animal Science and Technology, Yangzhou University, Yangzhou 225009, China; 3 Institutes of Agricultural Science and Technology Development, Yangzhou University, Yangzhou 225009, China; 4 College of Bioscience and Biotechnology, Yangzhou University, Yangzhou 225009, China; 5 Department of Animal & Avian Sciences, University of Maryland, College Park, MD 20742, USA; songj88@umd.edu; 6 Poultry Institute of Chinese Academy of Agricultural Sciences, Yangzhou 225003, China; hanwei830@163.com; 7 College of Biotechnology, Jiangsu University of Science and Technology, Zhenjiang 212100, China

**Keywords:** CRISPR/Cas9, chicken, sex chromosomes, genome engineering, gene insertion sites

## Abstract

The identification of accurate gene insertion sites on chicken sex chromosomes is crucial for advancing sex control breeding materials. In this study, the intergenic region NC_006127.4 on the chicken Z chromosome and the non-repetitive sequence EE0.6 on the W chromosome were selected as potential gene insertion sites. Gene knockout vectors targeting these sites were constructed and transfected into DF-1 cells. T7E1 enzyme cleavage and luciferase reporter enzyme analyses revealed knockout efficiencies of 80.00% (16/20), 75.00% (15/20), and 75.00% (15/20) for the three sgRNAs targeting the EE0.6 site. For the three sgRNAs targeting the NC_006127.4 site, knockout efficiencies were 70.00% (14/20), 60.00% (12/20), and 45.00% (9/20). Gel electrophoresis and high-throughput sequencing were performed to detect potential off-target effects, showing no significant off-target effects for the knockout vectors at the two sites. EdU and CCK-8 proliferation assays revealed no significant difference in cell proliferation activity between the knockout and control groups. These results demonstrate that the EE0.6 and NC_006127.4 sites can serve as gene insertion sites on chicken sex chromosomes for gene editing without affecting normal cell proliferation.

## 1. Introduction

It is widely acknowledged that the integration of exogenous genes into the recipient cell genome poses two major challenges including harmful effects and gene silencing [1]. The intricate composition of cellular structures further complicates the translation process of transgenes. CRISPR/Cas9-mediated targeted gene knock-in has become a conventional approach in transgenic technology. Selecting gene insertion sites that ensure stable and efficient expression of inserted genes without impeding the normal development of organisms is critical [2].

Friedrich and Soriano [3] discovered *Rosa26* gene insertion sites while investigating gene mutations in murine embryonic stem cells. Zambrowicz et al. [4] successfully inserted exogenous genes at this site, resulting in sustained and stable expression in murine individuals without compromising their growth and development. Subsequently, this site was demonstrated to exist in genomes of other animals such as pigs [5] and cattle [6], finding broad application in facilitating efficient gene editing through safe knock-in. Kotin [7] identified the AAVS1 site on the human autosomal chromosome, while Mali [8] achieved multi-gene editing at this site in human cells. Beyond well-known *Rosa26* and *AAVS1*, additional gene insertion sites exist in mammals, such as the porcine *β-actin* (*ACTB*) locus. Xiong [9] reported the pervasive presence of *ACTB* in porcine autosomes and subsequently, through CRISPR/Cas9-mediated homologous recombination, validated the safety of *ACTB* as a knock-in site for exogenous genes. At present, the application of gene insertion sites in mammalian gene editing has matured [10]. Ekaterina et al. [11] successfully inserted *EGFP* at the *β-actin* and *GAPDH* gene loci on chicken autosomes, demonstrating that these sites can serve as gene insertion sites for gene insertion at the cellular level. However, the majority of selected gene insertion sites in current gene editing studies are situated on autosomes, with scant reports on gene insertion sites on sex chromosomes.

Lee [12] identified a gene intergenic region (NC_006127.4) on the Z chromosome that permits the targeted knock-in of exogenous *GFP* genes. This enabled the production of transgenic primordial germ cells (PGCs) and subsequent transplantation led to the generation of transgenic chickens, offering initial insights into potential gene insertion sites for foreign gene insertion on the chicken sex chromosomes. Nevertheless, the corresponding gene insertion sites on the W chromosome need further investigation [13]. EE0.6 represents a non-repetitive sequence on the chicken W chromosome, encompassing a non-transcribed segment likely to be a pseudogene [14]. Building upon prior research, our study selected the intergenic region NC_006127.4 on the Z chromosome and the non-repetitive sequence EE0.6 on the W chromosome as prospective gene insertion sites for gene editing in chickens. Through the construction of gene knockout vectors targeting these two sites, we validated sgRNA knockout activity and assessed the impact of knockout sites on cell proliferation activity. We established the safety of these two sites and tested potential off-target effects of the sgRNAs in the knockout vectors, further corroborating the safety of our gene knockout system. This lays the foundation for developing a targeted knock-in system for gene editing on the chicken sex chromosomes. Our objective is to provide technical support for the insertion of exogenous functional genes, advance the exploration of chicken functional genomics, and contribute to the creation of transgenic animal models.

## 2. Materials and Methods

### 2.1. Sample Collection

The DF-1 chicken fibroblast line used in this experiment was purchased from the ATCC cell bank, and the Cas9/sgRNA-VK001-08 vector was constructed by Beijing Viewsolid Biotechnology Co., Ltd. (Beijing, China).

### 2.2. Construction of CRISPR/Cas9-Mediated Gene Knockout Vectors

CRISPR/Cas9-mediated gene knockout vectors were constructed by employing the online platform CRISPOR (http://crispor.tefor.net/, accessed on 16 June 2022) to design sgRNAs targeting the NC_006127.4 and EE0.6 sequences. Three sgRNA sequences with high scores and low off-target probabilities were selected for each target (Table 1). The target site sequences were subjected to BLAST comparison to ensure the specificity of the sgRNA sequences, thereby confirming their exclusive targeting of the intended loci without affecting unrelated gene regions. Subsequently, oligo primers were synthetically designed based on the selected sgRNA sequences, and these oligos underwent PCR annealing to yield double-stranded oligo sequences. The resulting oligo dimers were then integrated into the Cas9/sgRNA-VK001-08 gene knockout vector. After thorough homogenization of the double-stranded oligo with the Cas9/sgRNA vector, the ligation product was obtained following a 5 min incubation at 25 °C. The ligation product was added to competent cells (TSC-C14, Tsingke, Beijing, China) at a 1:10 ratio, gently mixed, and subjected to a 30 min ice bath. The transformed material was subsequently uniformly spread on an ampicillin-resistant agar plate using the “five-spot” technique and incubated overnight. Following a 12 to 16 h incubation period, single colonies were selected for sequencing. The sequencing primer used in this study was 5′-TGAGCGTCGATTTTTGTGATGCTCGTCAG-3′. Colonies with confirmed alignment through BLAST were subjected to expansion and agitation. High-purity plasmid DNA was extracted from the bacterial culture using the Endotoxin-Free Plasmid Maxi Kit (DP117, TIANGEN, Beijing, China) for large-scale purification.

### 2.3. In Vitro DF-1 Cell Subculture and Transfection

Healthy DF-1 cells were confirmed under the microscope by an elongated spindle-shaped morphology without vacuoles or filamentous structures. DF-1 cells were cultured in DMEM medium (30-2002, ATCC, Manassas, VA, USA) supplemented with 10% FBS (16000-044, Gibco, Waltham, MA, USA), and 1% penicillin–streptomycin–amphotericin B (PB180121, Pricella, Wuhan, China) and passaged when the cell growth density reached approximately 95%.

For transient transfection, DF-1 cells were evenly seeded into a 24-well cell culture plate at a density of 1.5 × 10^5^ cells per well. FugeneHD (E2311, Promega, Madison, WI, USA) was used for transfection according to the manufacturer’s instructions. Briefly, A solution containing 1 µg of plasmid and Opti-MEM (31985-047, Gibco, USA) and B solution consisting of 3 µL of FugeneHD and 47 µL of Opti-MEM were mixed gently and incubated at 37 °C for 15 min. The cells were washed with PBS, and a complete culture medium without triple antibiotics was added. After the incubation period, the transfection mixture was added to the cells followed by incubation at 39 °C and 5% CO_2_. The cell status and fluorescence expression were observed under the microscope after 24–48 h.

### 2.4. Extraction of Total RNA and Reverse Transcription

Upon collection of cell or tissue samples, TRNzol lysis reagent (DP424, TIANGEN, Beijing, China) was added in proportion to 5 × 10^6^ cells/mL or 50 mg animal tissue/mL. The homogenized samples were lysed at room temperature for 5 min, followed by the addition of 200 µL of chloroform (Sinopharm, Beijing, China). The mixture was vigorously vortexed for 15 s and then allowed to stand at room temperature for 3 min before being centrifuged at 4 °C, 12,000 rpm for 15 min. The aqueous phase was carefully transferred to a new RNase-free tube and an equal volume of isopropanol was added. After gentle inversion, the mixture was allowed to stand at room temperature for 10 min and then centrifuged at 4 °C, 12,000 rpm for 10 min, resulting in the appearance of a white pellet at the bottom of the tube. The supernatant was cautiously aspirated, and the pellet was washed with 75% ethanol. After centrifuging at 4 °C, 7500 rpm for 5 min, the supernatant was discarded, and the remaining liquid was gently removed after brief air-drying. Then, 20 µL of RNase-free water (10977-015, Gibco, USA) was added to the pellet, mixed gently, and incubated at 55 °C for 5 min to promote dissolution. After measuring the concentration, the RNA samples were stored at −80 °C for further use.

Total RNA from cells was reverse transcribed using the HiScript^®^ III RT SuperMix kit (R323-01, Vazyme, Nanjing, China). The obtained cDNA was diluted in 80 µL of RNase-free ddH_2_O to a final concentration of 10 ng/µL and stored at −20 °C for subsequent analysis.

### 2.5. qRT-PCR

According to the constructed knockout vector sgRNA/Cas9-VK001-08, detection primers VK-Cas9 were designed, and the chicken *ACTB* gene was used as the internal reference. Both upstream and downstream primers were designed for each target (Table 2). qRT-PCR technology was employed to examine the difference in Cas9 expression between the transfected DF-1 cells and normal cells at the RNA level. The qRT-PCR reaction system was composed of 20 μL: 10 μL of 2× ChamQ Universal SYBR qPCR Master Mix (Q711-02/03, Vazyme, Nanjing, China), 0.6 μL of upstream primer, 0.6 Μl of downstream primer, 2 μL of cDNA template, and RNase-free ddH_2_O to complete the reaction system to 20 μL. The reaction program consisted of 40 cycles at 95 °C for 10 s and 60 °C for 30 s. DF-1 cells were cultured with three biological replicates, and each sample was subjected to three technical replicates.

### 2.6. BCA Total Protein Quantification

In this study, BCA (CW0014S, Cwbio, Taizhou, Jiangsu, China) standard solutions were prepared by diluting in PBS to achieve the following gradient of concentrations: 2 μg/μL, 1 μg/μL, 0.5 μg/μL, 0.25 μg/μL, 0.125 μg/μL, 0.0625 μg/μL, and 0 μg/μL (blank) with a total of 7 concentrations. Then, a microplate assay (200 μL per well) was performed, and the working solution was prepared by mixing A solution and B solution at a volumetric ratio of 50:1, resulting in a greenish-blue color. Next, 25 μL of each freshly prepared standard solution and test sample were added to the 96-well plate, followed by the addition of 200 μL of BCA working reagent to each well. After thorough mixing, the plate was incubated at 37 °C for 30 min, followed by cooling to room temperature. The absorbance of each sample was measured at 562 nm using a spectrophotometer. A standard curve (with a confidence interval R^2^ ≥ 99%) was generated based on the concentration and absorbance values of the standard solutions. Using the standard curve, the total protein concentration of each sample was calculated.

### 2.7. Western Blot

Cell samples were collected, and protein extraction was performed using RIPA lysis buffer (R0010, Solarbio, Beijing, China). The protein concentration was determined following the method described in the section above. Western blot was then employed to detect the expression of Cas9 protein. Each sample was loaded with 20 μg of protein and mixed with SDS loading buffer. The protein samples were denatured at 100 °C for 10 min. An 8% SDS-PAGE gel (M00661, Genscript, Nanjing, China) was used for electrophoresis at ice-cold conditions. Subsequently, semi-dry transfer was performed using the following conditions: actin reference protein: 11 V for 23 min and Cas9 protein: 11 V for 23 min followed by 20 V for 27 min. The membrane was blocked with TBST containing 5% non-fat milk at room temperature for 2 h and then washed. Primary antibody was added and incubated overnight at 4 °C. The next day, the primary antibody (7A9-3A3, abcam, Eugene, OR, USA) was washed off, and the appropriate secondary antibody was added and incubated for 2 h. The signal was visualized using the ECL chemiluminescence method and captured using an imaging system.

### 2.8. T7E1 Enzyme Cleavage

After collecting the transfected cells at 48 h, they were resuspended in PBS buffer and subjected to cell sorting using the FITC channel. The sorted positive cells were centrifuged at 10,000 rpm for 1 min to obtain the cell pellet. After removing the supernatant, the genomic DNA was extracted using the Cell/Tissue Genomic DNA Extraction Kit (DP304, TIANGEN, Beijing, China). Simultaneously, untreated DF-1 cells were used as a control group, and the obtained genomic DNA was stored at −20 °C for later use.

Based on the ability of the T7E1 enzyme to recognize and cleave incompletely matched DNA sequences in the genome, it can be utilized as a method to detect CRISPR/Cas9-mediated gene knockout. In the experiment, primers were designed to amplify regions of approximately 200–250 bp before and after the knockout target sequence (Table 3). The PCR reaction system was set at 10 μL: 2× PrimerStar Max, 1 μL of upstream primer, 1 μL of downstream primer, 100 ng of genomic DNA, and RNase-free ddH_2_O to complete the reaction system to 10 μL. The amplification reaction program included 95 °C for 3 min, 35 cycles of 95 °C for 15 s, 55 °C (for EE0.6)/57 °C (for NC_006127.4) for 15 s, and 72 °C for 30 s, with a final extension at 72 °C for 5 min.

The obtained products were cloned and subjected to 2% agarose gel electrophoresis for targeted fragment recovery. The specific procedures are detailed in the DNA purification recovery kit manual (DP214, TianGen, Beijing, China). After recovery, the fragments were denatured and annealed. Subsequently, 0.5 μL of T7E1 enzyme (M0302L, NEB, Ipswich, MA, USA) was added to each reaction tube. After 30 min of enzyme digestion at 37 °C, 2 μL of DNA Loading Buffer was added and mixed by gentle pipetting. The reaction was terminated at 65 °C for 10 min. The PCR products were subjected to gel electrophoresis, and the results of the enzyme digestion were observed under a UV spectrophotometer. The knockout efficiency was calculated as (mutant band gray value/total gray value of mutant and non-mutant bands) × 100%.

### 2.9. TA Cloning

The PCR-amplified target bands obtained as mentioned above were ligated to the T-vector (pClone007 Versatile Simple Vector, Takara Bio, Beijing, China) at 25 °C for 5 min to obtain the ligated products. The ligation reaction mixture consisted of 2 μL of 5× pClone007 Versatile Simple Vector Mix, 25 ng of PCR-amplified product, and RNase-free ddH_2_O to complete the reaction system to 10 μL. Subsequently, 5 μL of the ligation product was added to 50 μL of competent cells for transformation, followed by overnight incubation. After colony picking and sequencing, the actual knockout types were determined by performing BLAST alignment with the wild-type sequence, and the actual TA cloning knockout efficiency was calculated. The knockout efficiency was determined as the percentage of colonies with sequence mutations over the total number of sequenced colonies, multiplied by 100%.

### 2.10. SSA In Vitro Activity Assay

According to the principle of in vitro SSA activity assay, the target site sequence was inserted into the Luciferase reporter vector together with the terminator, resulting in the loss of Luciferase fluorescence activity at the target site. Cas9 and sgRNA recognize and cleave the target site, leading to DNA double-strand breaks. Subsequently, a homology-directed repair mechanism can restore the Luciferase fluorescence activity. The constructed VK001-08 gene knockout vectors for the two target sites, along with the donor vectors and the constructed Luciferase recombinant reporter gene vector, were co-transfected. After 48 h, samples were collected, and the Luciferase luminescence activity expression was detected using the Dual-Luciferase Reporter Assay System (DL101-01, Promega, Madison, WI, USA).

### 2.11. The CCK-8 Cell Viability

After transfection, DF-1 cells were seeded in a 96-well plate at a density of 1 × 10^4^ cells per well. The cells were pre-cultured for 24 h before adding 10 μL of CCK-8 Solution (A311-01, NovoPro, Shanghai, China) to each well. The plate was gently shaken to ensure thorough mixing and then incubated for 2 h in a cell culture incubator. The absorbance at 450 nm was measured using an enzyme-linked immunosorbent assay (ELISA) reader, and the cell number changes were calculated based on a standard curve. This CCK-8 cell viability assay allowed us to assess the proliferation and viability of DF-1 cells after 24 h of transfection under the specific experimental conditions.

### 2.12. EdU Cell Proliferation Assay

Cell proliferation capacity was accessed using the EdU Cell Proliferation Assay Kit manual (C10310-2, RiboBio, Guangzhou, China) according to the manufacturer’s instruction 48 h after transfection. Following staining, the cells were immediately observed under fluorescence microscopy and photographed. Cells showing overlapping signals of Apollo643 staining and Hoechst staining were considered EdU-positive.

### 2.13. Potential Off-Target Effects Were Assessed Using Agarose Gel Electrophoresis and Sequencing to Detect Off-Target Sites

Regarding the sgRNA sequences targeting the EE0.6 and NC-006127.4 loci, we utilized the website (http://www.rgenome.net/cas-offinder/, accessed on 9 August 2023) to predict potential off-target sites. Based on the scores provided by the website, we selected six potential off-target sites for EE0.6-sg1 (refer to Table 4) and five potential off-target sites for NC4-sg1 (refer to Table 5). To further validate the actual off-target activity of these potential sites, we designed forward and reverse primers flanking the off-target sites (refer to Table 6). These primers were used for PCR amplification with cDNA templates obtained from Section 2.4. Subsequently, the PCR products of the expected size were analyzed via 1% agarose gel electrophoresis to confirm the presence of a single band. Bands of the expected size were excised from the gel, purified, and subjected to sequencing. 

### 2.14. Data Analysis

All experimental data are presented as mean ± standard error. The data were organized using Microsoft Excel 2016 and analyzed for significance and plotted using GraphPad Prism 6 software. Significance analysis was performed using Student’s *t*-test and one-way ANOVA to determine the significance of differences between data groups (* *p* < 0.05, significant difference; ** *p* < 0.01, extremely significant difference). 

## 3. Results

### 3.1. Construction of CRISPR/Cas9-Mediated Knockout Vectors Targeting the EE0.6 and NC_006127.4 Loci, and Validation of Their Knockout Activity

**Figure 1:**
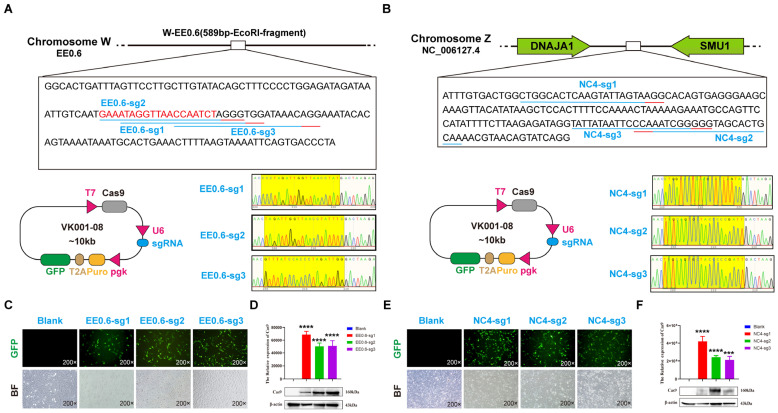
(**A**,**B**): Sequence diagram of three different sgRNAs designed according to EE0.6 and NC_006127.4 loci, vector connection schematic diagram, and sequencing peak diagram. (**C**,**E**): Fluorescent expression of EGFP transfected DF-1 cells with vector (control group: blank; experimental group: sg1, sg2, sg3, all with EGFP fluorescence expression, 200×). (**D**,**F**): qRT-PCR detection of relative expression of Cas9 (the expression of Cas9 mRNA in the experimental group is significantly higher than that in the control group, *** *p* < 0.01, **** *p* < 0.001); Western blot detection of Cas9 protein expression (Cas9 protein expression exists in the experimental group, Cas9: 160 KD; actin: 43 KD).

Based on the sequences of EE0.6 and NC_006127.4 obtained from the NCBI database, we utilized the CRISPOR online design platform (http://crispor.tefor.net/, accessed on 16 June 2022) to design and select three specific sgRNAs targeting each locus. Subsequently, we designed oligo annealing primers based on the target sequences, and after annealing the forward and reverse strands, they were ligated into the VK001-08 vector to construct EE0.6-sgRNA/Cas9 and NC_006127.4-sgRNA/Cas9 gene knockout vectors. Sequencing results confirmed the successful incorporation of sgRNA1, sgRNA2, and sgRNA3 for both loci into the vectors, denoted as EE0.6-sg1, EE0.6-sg2, and EE0.6-sg3 and NC4-sg1, NC4-sg2, and NC4-sg3 (Figure 1A,B).

To assess and compare the knockout efficiency of the constructed NC_006127.4-Cas9/sgRNA vectors, the three vectors targeting each of the two loci were individually transfected into DF-1 cells using lipofection. After 48 h of transfection, the expression of green fluorescence was observed within the cells (Figure 1C,E). qRT-PCR results revealed a significant increase in Cas9 expression in the experimental group cells compared with the control group cells (*p* < 0.01). Concurrently, Western blot analysis detected the presence of Cas9 protein expression in the transfected cells, confirming that EE0.6-Cas9/sgRNA vectors could express in DF-1 cells (Figure 1D,F).

### 3.2. In Vitro Validation of Gene Knockout Efficiency at the EE0.6 and NC_006127.4 Loci

**Figure 2:**
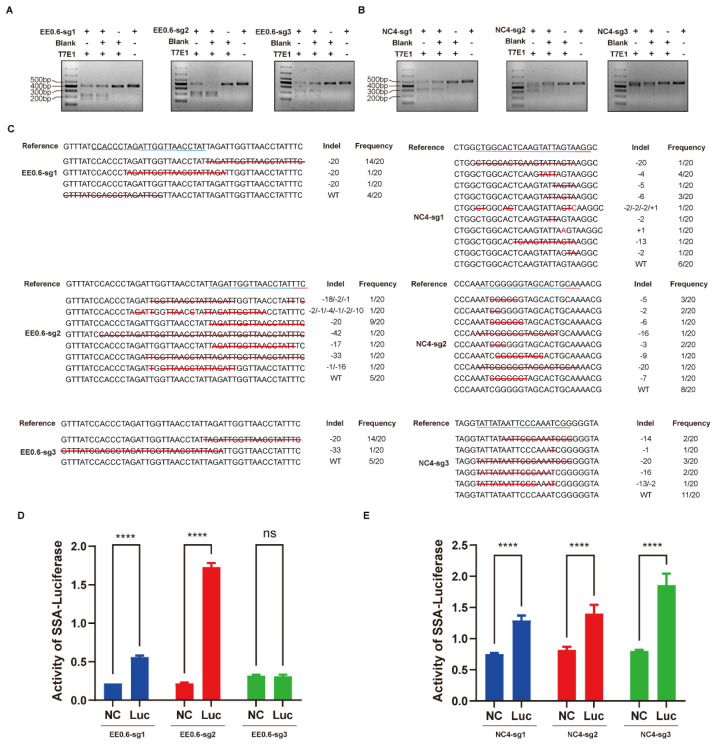
(**A**,**B**): T7E1 enzyme cleavage detection of sgRNA cleavage efficiency on EE0.6 and NC_006127.4 (sg1, sg2, and sg3 all have cleavage activity, Marker: DL1000). (**C**): Actual mutation type of TA cloning sequence (reference: sgRNA sequence, the blue underline is the sgRNA sequence, and the red is the PAM sequence; WT: wild type; Indel: actual number of base changes; Frequency: frequency of mutations). (**D**,**E**): Luciferase SSA reporter vector detection of luciferase activity (the sg1 and sg2 groups have significantly higher fluorescent activity than the control group on the EE0.6 site, and there is no significant difference between the sg3 and the control groups because of lower mutation efficiency; the sg1 and sg3 groups have significantly higher fluorescent activity than the control group on the NC_006127.4 site, and the sg2 group has significantly higher fluorescent activity than the control group, **** *p* < 0.001).

Transfected cells from each group were collected and subjected to the T7E1 enzyme cleavage assay, revealing cleavage activity at the target sequences of all six sgRNAs for both EE0.6 and NC_006127.4 loci (Figure 2A,B). Analysis of the band intensities of the cleaved fragments indicated that the knockout efficiencies of the three sgRNA/Cas9 vectors at the EE0.6 locus were 48.90%, 52.13%, and 39.40%, respectively. Likewise, at the NC_006127.4 locus, the knockout efficiencies of the three sgRNA/Cas9 vectors were 44.70%, 47.29%, and 45.85%, respectively. To validate the actual in vitro knockout efficiency, a TA cloning experiment was conducted, with 20 individual colonies picked from each group. Sequencing results demonstrated gene knockout efficiencies for the EE0.6 locus of 80.00% (16/20), 75.00% (15/20), and 75.00% (15/20), and for the NC_006127.4 locus of 70.00% (14/20), 60.00% (12/20), and 45.00% (9/20). The specific mutation types are detailed in the figure below (Figure 2C).

For external validation of the knockout efficiency, a Luciferase-SSA reporter vector was constructed, and the luminescence assay results indicated that at the EE0.6 locus, the luminescence activity of the sg1 and sg2 vectors was significantly higher than the control group (** *p* < 0.01). At the NC_006127.4 locus, the luminescence activity of the sg2 group was significantly higher than the control group, while the sg1 and sg3 groups exhibited marginally higher luminescence activity than the control group (** *p* < 0.01) (Figure 2D).

Taken together, the results demonstrate that all three sgRNA/Cas9 vectors targeting each of the two loci possess knockout activity. Combining this with the actual knockout efficiency from the TA cloning experiment, it is evident that the knockout efficiency of sgRNA1 and sgRNA2 vectors is superior to that of sgRNA3 vectors at both loci.

### 3.3. In Vitro Validation of Gene Editing Safety at the EE0.6 and NC-006127.4 Loci

**Figure 3:**
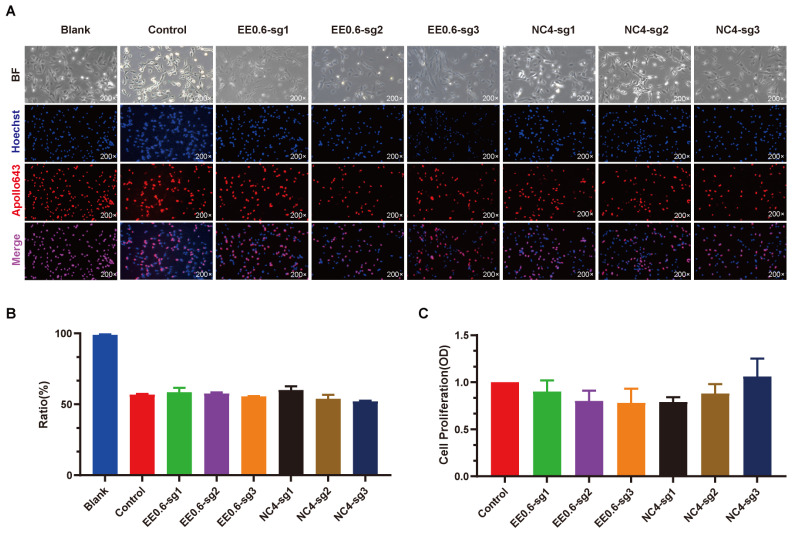
(**A**): EdU staining was used to detect the proliferative ability of DF-1 cells with EE0.6 and NC_006127.4 loci knocked out (200× magnification). (**B**): Bar graph showing the results of EdU staining for the proliferative ability of DF-1 cells with EE0.6 and NC_006127.4 loci knocked out. The ratio (%) was calculated as the number of Apollo643-stained cells divided by the number of Hoechst33342-stained cells. In comparison to the control group, there was no significant difference in the proliferative activity of the experimental group cells. (**C**): The CCK-8 assay was used to measure the viability of DF-1 cells with EE0.6 and NC_006127.4 loci knocked out. The proliferative activity of the experimental group cells, compared to the control group, showed no significant difference.

To assess whether the knockout of the EE0.6 and NC_006127.4 loci has any impact on the normal growth and proliferation of cells, a proliferation capability assay was conducted on DF-1 cells transfected with knockout vectors targeting these two sites. The EdU labeling assay results indicated that the proliferation activity of cells following the knockout of EE0.6 and NC_006127.4 was not significantly different compared to the control group (Figure 3A,B). Likewise, the CCK-8 assay results corroborated the EdU labeling results, demonstrating that the proliferation activity of cells after transfection with knockout vectors was not significantly altered compared to the control group (Figure 3C). These results collectively suggest that the knockout of the EE0.6 and NC_006127.4 loci does not adversely affect the normal proliferative capacity of cells. Consequently, these sites can be considered safe gene insertion sites for the insertion of exogenous target fragments, providing a foundational basis for establishing targeted knock-in systems on the two loci of the chicken sex chromosomes.

### 3.4. Detection of Off-Target Effects of the EE0.6 and NC_006127.4 Knockout Vectors

**Figure 4:**
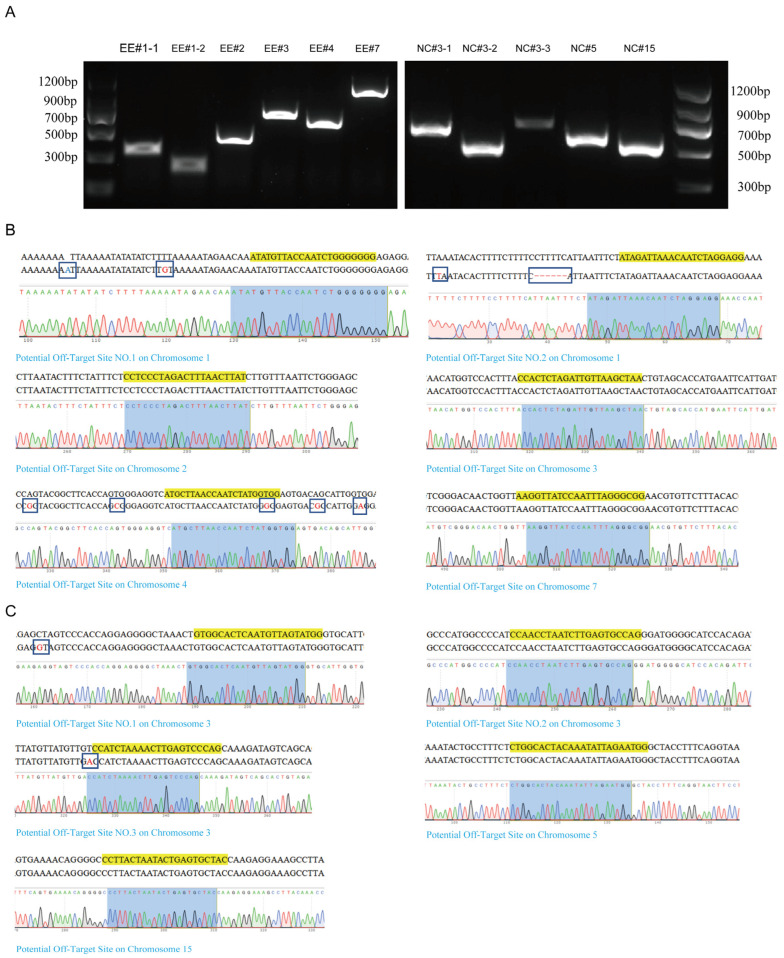
(**A**): Agarose gel(1%) electrophoresis to detect potential off-target site primer specificity (# is followed by the number of the predicted off-target site). (**B**): Sanger sequencing of EE0.6-sg1 potential off-target sites (blue letters represent base substitution, red represents base increase—represents base deletion, and yellow highlights are potential off-target site sequences). (**C**): Sanger sequencing of NC4-sg1 potential off-target sites (blue letters represent base substitution, red represents base increase—represents base deletion, and yellow highlights are potential off-target site sequences).

In order to detect the potential off-target effects of the EE0.6 and NC_006127.4 loci and their corresponding sgRNA knockout vectors, ensuring that the knockout of the target sites does not affect the expression of genes in other genomic regions, a series of experiments were conducted. Predicted off-target site regions, both upstream and downstream of the target sequences, were amplified using PCR with designed primers. The resulting PCR products were subjected to 1% agarose gel electrophoresis, revealing a single band of the expected size (Figure 4A), indicating the specificity of the designed primers for the predicted off-target sites. The specific bands were excised from the gel, and the final purified products were subjected to sequencing.

The sequencing results indicated that out of the six potential off-target sites in the genome for the EE0.6-sg1 knockout vector, three sites exhibited minimal base mutations near their locations (Figure 4B). These mutations were all within intronic regions of the chromosomes. None of the other potential off-target sites showed any base mutations. Similarly, among the five potential off-target sites for the NC4-sg1 knockout vector, two sites had minor base mutations near their locations, again within intronic regions, while no base mutations were detected in the other potential off-target sites (Figure 4C).

These results demonstrate that the designed sgRNA knockout vectors targeting the EE0.6 and NC_006127.4 loci do not exhibit significant off-target effects. They can achieve specific knockout effects safely, without affecting the expression of genes in similar regions of the genome. This provides strong support for the subsequent introduction of functional genes into the chicken ZW chromosomes.

## 4. Discussion

In the construction of gene knock-in systems, achieving precise knockout at gene insertion sites is an indispensable factor for the successful insertion of exogenous genes. Among existing gene editing technologies, CRISPR/Cas9-mediated gene knockout stands out because of its simplicity in design and ability to achieve targeted gene disruption, making it a primary molecular tool for building gene knock-in systems [15]. Lee et al. [16] demonstrated the expression of CRISPR/Cas9 knockout vectors in DF-1 cells through qRT-PCR and confirmed vector activity through in vitro dual luciferase reporter assay. Cheng et al. [17] analyzed the types of base mutations generated by CRISPR/Cas9 knockout vectors at target sites in DF-1 cells using T7E1 enzyme cleavage and TA cloning sequencing, thereby validating vector knockout activity in vitro. Doran et al. [18] utilized Sperm Transfection-Assisted Gene Editing (STAGE) and the CRISPR/Cas9 system to obtain embryos with biallelic and *DMRT1* gene mutations, with efficiencies ranging from 0% to 26%.

In this study, CRISPR/Cas9 technology was also employed, utilizing the VK001-08 vector as a framework to design and construct gene knockout vectors for the EE0.6 locus on the chicken W chromosome and the NC_006127.4 locus on the Z chromosome. Following transfection into DF-1 cells, the constructed sgRNA/Cas9 vectors were verified to knockout the target site sequences effectively, and specific types of base mutations resulting from the knockout were identified through sequencing. The results demonstrate that the selected three specific sgRNA sequences exhibit knockout activity, enabling directed knockout at gene insertion sites. 

The selection of gene insertion sites on the genome is a prerequisite for achieving targeted insertion of exogenous genes. Gene insertion sites, as insertion sites for foreign genes, need to fulfill two conditions. Firstly, knocking out these sites should not affect the normal growth, development, or reproduction of individuals; secondly, they should ensure stable and normal expression of the inserted foreign fragments. Zhang et al. [19] utilized the CRISPR/Cas9 system to establish a transgenic crab-eating macaque model and found that crab-eating macaques lacking *SIRT6* exhibited severe developmental delay during the perinatal period, indicating the crucial role of *SIRT6* in their growth and development. Browning et al. [20] discovered that inserting the human CD1A promoter and coding region into the *Hipp11* intergenic region on mouse chromosome 11 allows efficient integration of exogenous fragments. Because of its location between two genes, the impact of inserted foreign genes on endogenous genes is minimized, making *Hipp11* a noteworthy gene insertion site.

Goodwin et al. [21] found that inserting foreign genes at certain sites in the mouse genome can disrupt the original gene sequences, leading to phenotypic consequences in individuals. Therefore, it is essential to identify and screen for gene insertion sites that are silenced and have a low mutation rate during animal gene editing. In animal cells, gene knockout or knock-in is a vital approach for genome editing in individuals. Especially for avian species like chickens, for which it is difficult to generate transgenic offspring because of their unique reproductive physiology, achieving higher efficiency in gene knockout or knock-in in primordial germ cells or pluripotent stem cell lines and purifying them for injection into chicken embryos is essential. In our previous work, a gene knockout system mediated by CRISPR/Cas9 was established, and a knockout vector for the chicken C2EIP gene was constructed, demonstrating an efficiency of approximately 27% in both in vitro and in vivo assays [22]. 

Furthermore, when performing gene knockout, the off-target effects commonly associated with the CRISPR/Cas9 system need to be considered. With an sgRNA length of around 20 nt, many sequences in the entire genome differ by only one or two nucleotides from the target sequence. As a result, sgRNAs can easily target similar sites, recruiting the Cas9 protein for cleavage and potentially leading to silencing of non-target genes, thereby affecting gene editing [23]. Therefore, predicting and screening potential off-target sites for the designed sgRNA and detecting off-target effects are crucial.

In this study, we selected the intergenic region NC_006127.4 on chromosome Z and the EE0.6 region on chromosome W as candidate gene insertion sites for exogenous fragment insertion and conducted safety validation. Different from the Z chromosome, the W chromosome has fewer functional genes, and there is limited research on gene insertion sites on the chicken W chromosome. EE0.6, as a non-repetitive DNA sequence highly conserved in birds, is often used as a sex-specific marker gene for avian gender determination [24], yet its functional significance remains unreported. Therefore, in this study, we selected the EE0.6 site and investigated its safety after knockout. To assess the feasibility of candidate gene insertion sites for knockout and to select the sgRNA with the highest knockout efficiency for subsequent in vitro validation, we designed three sgRNA/Cas9 vectors for each of the EE0.6 and NC_006127.4 sites. The in vitro knockout efficiency of the three sgRNA/Cas9 vectors targeting the EE0.6 site was approximately 80.00%, 75.00%, and 75.00%, respectively. For the three sgRNA vectors targeting the NC_006127.4 site, the in vitro knockout efficiencies were approximately 70.00%, 60.00%, and 45.00%, respectively. Subsequently, the sgRNA1 with the highest in vitro knockout efficiency for each candidate gene insertion site was selected for potential off-target site prediction and sequencing analysis. The results indicated that the in vitro knockout efficiency achieved was comparable to levels required for gene editing. Combined with the observation that knockout of EE0.6 and NC_006127.4 did not affect the proliferative activity of DF-1 cells, these sites possess the characteristics of gene insertion sites. The sgRNA knockout vectors showed no apparent off-target effects, suggesting that these two sites can be considered candidate insertion sites on the chicken sex chromosomes for subsequent in vivo validation and the study of inserting functional genes, thus establishing a gene editing model utilizing chicken sex chromosomes as a platform for gene expression.

## 5. Conclusions

The constructed sgRNA/Cas9 knockout vectors targeted to EE0.6 and NC_006127.4 exhibit normal expression in DF-1 cells and both possess knockout activity. The highest knockout efficiency achieved for the EE0.6 locus was 80.00%, while the NC_006127.4 locus reached a maximum efficiency of 70.00%. Both EE0.6 and NC_006127.4 can serve as gene insertion sites for gene knockout on chicken sex chromosomes. Notably, the knockout of these two loci does not affect the normal proliferative activity of the cells.

## Figures and Tables

**Table 1:** Sequences of sgRNA targeting site.

Sample	sgRNA + *PAM*
NC_006127.4-sg1	CTGGCACTCAAGTATTAGTA *AGG*
NC_006127.4-sg2	TTGCAGTGCTACCCCCGATT *TGG*
NC_006127.4-sg3	TATTATAATTCCCAAATCGG *GGG*
EE0.6-sg1	ATAGGTTAACCAATCTAGGG *TGG*
EE0.6-sg2	GAAATAGGTTAACCAATCTA *GGG*
EE0.6-sg3	CCAATCTAGGGTGGATAAAC *AGG*

**Table 2:** Primers of qRT-PCR.

Primers	Sequence (5′ to 3′)
*VK-Cas9*-F	ATCGCCACAGCATA
*VK-Cas9*-R	CATCCACCTTAGCC
*β-actin*-F	CAGCCATCTTTCTTGGGTAT
*β-actin*-R	CTGTGATCTCCTTCTGCATCC

**Table 3:** Sequence of T7E1 digestion target fragment amplification.

Sample	Primer Sequences (5′ to 3′)
NC_006127.4-F	GGCACGCATCCACTCTGTACAGAG
NC_006127.4-R	CTCCAGGACCAGGGCATTCCT
EE0.6-F	AGTGCTTCAGCTGGACTTCA
EE0.6-R	TGGTCCTATGCCTACCACAT

**Table 4:** Potential off-target sites for EE0.6-sg1.

Bulge Type	crRNA	DNA	Chromosome	Position
RNA	ATAGGTTAACCAATCTAGGGNGG	ATAtGTT-ACCAATCTgGGGGGG	1	144,871,693
RNA	ATAGGTTAACCAATCTAGGGNGG	ATAGaTTAAaCAATCTA-GGAGG	1	153,589,494
DNA	ATAGGTTAACCAA-TCTAGGGNGG	ATAaGTTAACaAAGTCTAGGGAGG	2	11,394,848
RNA	ATAGGTTAACCAATCTAGGGNGG	tTAGcTTAA-CAATCTAGGGTGG	3	80,803,156
RNA	ATAGGTTAACCAATCTAGGGNGG	AT-GcTTAACCAATCTAtGGTGG	4	20,449,610
RNA	ATAGGTTAACCAATCTAGGGNGG	A-AGGTTAtCCAATtTAGGGCGG	7	18,245,507

**Table 5:** Potential off-target sites for NC4-sg1.

Bulge Type	crRNA	DNA	Chromosome	Position
RNA	CTGGCACTCAAGTATTAGTANGG	gTGGCACTCAA-TgTTAGTATGG	3	7,365,457
RNA	CTGGCACTCAAGTATTAGTANGG	CTGGCACTCAAG-ATTAGgtTGG	3	85,597,875
RNA	CTGGCACTCAAGTATTAGTANGG	CTGGgACTCAAGTtTTAG-ATGG	3	71,589,675
DNA	CTGGCACT-CAAGTATTAGTANGG	CTGGCACTACAAaTATTAGaATGG	5	33,683,785
RNA	CTGGCACTCAAGTATTAGTANGG	gTaGCACTC-AGTATTAGTAAGG	15	5,984,301

**Table 6:** Primers of off-target sites.

Primer	Sequence(5′ to 3′)	Base Number
EE#1-1-F	CACTCGTATAAGAACCATGA	20
EE#1-1-R	GGGAGGTAAATCTAAACTGT	20
EE#1-2-F	ACCTACGTGATATTGATAGC	20
EE#1-2-R	GGTGATAGCAGAGGATACTA	20
EE#2-F	GCCAATACCGATGACTCT	18
EE#2-R	GGAAGTAGAATAGCACCTAAG	21
EE#3-F	CACTCTGCTGTAGTTGAT	18
EE#3-R	CTGCTTCTGACGCTATC	17
EE#4-F	TGCTGGTCAGTTCTTGTTCT	20
EE#4-R	CGCCTGGACATGGAATCA	18
EE#7-F	CTCGGTGACAAGGTGGTT	18
EE#7-R	CACATATCACTGCCATCCTATA	22
NC#3-1F	AATGTAGGTCAGGTGAAGTT	20
NC#3-1R	GTGTTATTGGAGTTGTGTCA	20
NC#3-2F	TTTGCAGGTGTCTCTATTTG	20
NC#3-2R	CCTCAGTATAGTCTCTGGTATA	22
NC#3-3F	ACTCTGCTCTAGGTGTTC	18
NC#3-3R	CTCTTGTGCTAAGGTGTATT	20
NC#5-F	GGAGTATTACCTTCTTCACA	20
NC#5-R	CGTAGATGAGTTGGCTAG	18
NC#15-F	TAGTCATAAGTGTCCTTGGT	20
NC#15-R	CTGTGATTGAGAGCATCTG	19

## Data Availability

The data that support the findings of this study are available from the corresponding author upon reasonable request.
